# Short hairpin RNA targeting 2B gene of coxsackievirus B3 exhibits potential antiviral effects both *in vitro* and *in vivo*

**DOI:** 10.1186/1471-2334-12-177

**Published:** 2012-08-06

**Authors:** Hailan Yao, Yangde Zhang, Feng He, Caihong Wang, Zonghui Xiao, Jizhen Zou, Fang Wang, Zhewei Liu

**Affiliations:** 1 Molecular Immunology Laboratory, Capital Institute of Pediatrics, YaBao Road 2, Beijing, 100020, China; 2 National Hepatobiliary and Enteric Surgery Research Center of Ministry of Health, Central South University, Xiangya Road 87, Changsha, Hunan, 410008, China

**Keywords:** Coxsackievirus, Myocarditis, shRNA, Lentivirus vector

## Abstract

**Background:**

Coxsackievirus B3 is an important infectious agent of viral myocarditis, pancreatitis and aseptic meningitis, but there are no specific antiviral therapeutic reagents in clinical use. RNA interference-based technology has been developed to prevent the viral infection.

**Methods:**

To evaluate the impact of RNA interference on viral replication, cytopathogenicity and animal survival, short hairpin RNAs targeting the viral 2B region (shRNA-2B) expressed by a recombinant vector (pGCL-2B) or a recombinant lentivirus (Lenti-2B) were tansfected in HeLa cells or transduced in mice infected with CVB3.

**Results:**

ShRNA-2B exhibited a significant effect on inhibition of viral production in HeLa cells. Furthermore, shRNA-2B improved mouse survival rate, reduced the viral tissues titers and attenuated tissue damage compared with those of the shRNA-NC treated control group. Lenti-2B displayed more effective role in inhibition of viral replication than pGCL-2B *in vivo*.

**Conclusions:**

Coxsackievirus B3 2B is an effective target of gene silencing against coxsackievirus B3 infection, suggesting that shRNA-2B is a potential agent for further development into a treatment for enterviral diseases.

## Background

Coxsackievirus B3 (CVB3) is among the most common and significant causative agents of heart muscle disease in human. CVB3 is a member of the genus *Enterovirus*, which belongs to the family *Picornaviridae*. The spectrum of disease caused by these viruses ranges from very mild to life-threatening [1]. In some cases, CVB3 may escape the intestinal tract to produce serious diseases, such as viral myocarditis [2], pancreatitis [3] and aseptic meningitis [4]. It is estimated that at least 50% of the cases of infection-caused heart diseases are attributable to CVB3 infection [5]. However, there is currently no available vaccine and no specific drug to eliminate virus infection.

The CVB3 genome is a single-stranded positive-sense RNA molecule of approximately 7.4 kb. It contains a 5’-untranslated region (5’-UTR), a single long open reading frame, 3’-UTR, and a poly(A) tail. The four capsid proteins (VP1-4) and seven nonstructural proteins (2A, 2B, 2C, 3A, 3B, 3C, 3D) are generated by viral protease-mediated process of the single polyprotein translated from the genomic RNA [6]. CVB3 replication induces a direct cytopathic effect (CPE) on infected cells and direct tissue damage in various animal models [7,8]. Therefore, it is considered that virus elimination at a specific target can be the key therapeutic strategy to treat or attenuate CVB3-related disease.

RNA interference (RNAi) is a mechanism that inhibits specific gene expression by using short double-stranded RNA (dsRNA), which is converted to small interfering RNA (siRNA) as the active agent. RNAi is now employed as a potential therapeutic method against pathogenic viruses [9,10]. It is reported that several human pathogens, including human immunodeficiency virus [11], the hepatitis B [12] and C [13] viruses, influenza A virus [14] and coxsackievirus [15], were inhibited or eliminated by RNAi.

In our previous study, we designed twelve siRNAs targeting distinct regions of the CVB3 genome, and investigated their antiviral activities in HeLa cell [16]. The most effective one is siRNA-2B, targeting viral RNA at 2B region (3753-3771), achieving a 90% reduction of CVB3 replication [17]. In this study, we developed a recombinant plasmid and a lentivirus vector that delivered the short hairpin RNAs (shRNAs) targeting 2B gene (3753-3771) in cell culture system and in an animal model to evaluate the impact of shRNA-2B on CVB3 replication, pathogenicity and survival *in vitro* and *in vivo*.

## Methods

### Cell culture, virus, transfection and infection

HeLa and 293T cells were maintained in Dulbecco’s modified Eagle’s medium (DMEM) supplemented with 10% fetal bovine serum, 100 units/ml penicillin, and 100 μg/ml streptomycin. CVB3M strain was amplified in HeLa cells by infection. This strain was a kind gift from Dr S.A. Huber, University of Vermont, U.S.A. [18]. To assess the validity of shRNA-2B, HeLa cells were seeded into 12-well plates (1 × 10^5^ cells/well) overnight. When reached 50-60% confluency, cells were washed with phosphate buffered saline (PBS) and transfected with plasmid or transduced with lentivirus. At 24 h post transfection or 48 h post transduction, cells were washed and infected with CVB3 (MOI = 0.01) for 1 h. The cells were then overlaid with complete medium and incubated at 37°C with 5% CO_2_. At different time points post infection, supernatants were collected to determine the virus titers by viral plaque assay. Experiments were carried out 3 times.

### Recombinant plasmid and lentivirus generation

pHelper 1.0, pHelper 2.0 and pGCSIL-GFP plasmids were purchased from Shanghai GeneChem Co. Ltd. (Shanghai, China). To construct the recombinant vector, RNAi stem-loop DNA oligos containing the target sequences (GGACTATGTGGAACAGCTT) in the region of CVB3 2B were chemically synthesized, annealed and cloned into the *Age*I/*Eco*RI-digested pGCSIL-GFP, thus generating the plasmid pGCL-2B. The lentiviral vector pGCSIL-GFP contained a U6 promoter to express continuously the shRNA and CMV promoter to express the green fluorescent protein (GFP), which could be used to detect the transfection efficiency of viral packaging and infection. Recombinant lentiviruses were generated by co-transfection of 293T cells with 20 μg pGCL-2B and packaging vectors (15 μg pHelper1.0, 10 μg pHelper2.0) using 100 μl of Lipofectamine 2000™ reagent according to the manufacturer’s instructions (Invitrogen, USA). The recombinant lentivirus was designated Lenti-2B. The viral supernatant was harvested 48 h after transfection, passed through 0.45 μm filters and concentrated. The viral titer was determined by infecting 293T cells with serial dilutions of concentrated lentivirus, and then determining the GFP expression of infected cells by fluorescence microscopy 96 h after infection. Therefore, the titer is expressed as “transduction unit (TU)/ml”. A scramble siRNA sequence (5’-UUCUCCGAACGUGUCACGU-3’) was used to generate the non-silence control plasmid and lentivirus that were designated pGCL-NC and Lenti-NC.

### Short hairpin RNAs treatment *in vivo*

6-week-old BALB/c male mice were injected via caudal vein with plasmid or lentivirus. Entranster™-in vivo transfection reagent (Engreen Biosystem Co, Ltd. China) was used to deliver the plasmids. Control mice were injected with pGCL-NC, Lenti-NC and minimum essential medium (MEM), respectively. After 24 h, all mice were inoculated intraperitoneally with a dose of 8 × 10^3^ plaque-forming units (pfu)/mouse of CVB3 virus. Some mice were observed for survival time. Other mice were euthanized on days 3, 5 and 7 after infected with CVB3 (n = 10 per group). Experiments were carried out 3 times. The animal experiment has been performed with the approval of the ethics committee of Capital Institute of Pediatrics and followed internationally recognized guidelines.

### Organ virus titers and histological analysis

Hearts and pancreases from mice were weighted, homogenized in 0.5 ml MEM, and centrifuged for 10 minutes at 1000 rpm. In the supernatants, infectious virus was analyzed by viral plaque assay, and the viral titer in infected tissue was expressed as pfu/g. To assess severity of myocarditis and pancreatitis, paraffin-embedded sections of tissues were stained with hematoxylin-eosin and examined histopathologically for evidence of inflammation and necrosis. The immunohistochemistry staining for CVB3 and GFP were performed using anti-coxsackievirus B3 monoclonal antibody (Chemicon International, USA) and anti-GFP monoclonal antibody (Chemicon International, USA) as the primary antibody following the procedure described previously [19].

### Viral plaque assay

HeLa cells were seeded into six-well plates (4 × 10^5^ cells/well) and incubated at 37°C with 5% CO_2_ for 48 h. When cell confluency reached approximately 90%, cells were washed with PBS to remove the fetal bovine serum and then overlaid with 200 μl of 1:10 diluted supernatant. The cells were incubated at 37°C for 60 min and the supernatants were removed, and washed with PBS. Finally, cells were overlaid with 2 ml of sterilized soft Bacto-agar/MEM (2 × MEM:1.5% Bacto-agar = 1:1). The cells were incubated at 37°C for 72 h, fixed with Carnoy’s fixative for 30 min and then stained with 1% crystal violet. The plaques were counted and the viral pfu/ml was calculated [20]. Each sample was tested in triplicate.

### Statistics

All statistical analyses were performed using the SPSS 11.5 computer software program. Survival was analyzed using Log-rank (Mantel-Cox) method. The significance of variability among the experimental groups was determined by Mann–Whitney U test. All differences were considered statistically significant at *P* < 0.05.

## Results

### Construction of recombinant plasmid and lentivirus expressing shRNA targeting CVB3

In this study, recombinant plasmid expressing the shRNA targeting 2B sequence was cloned into pGCSIL-GFP and named pGCL-2B. This plasmid was used to pack the infectious lentivirus particles by co-transfection of 293T cells with other packaging plasmids. The lentivirus was named Lenti-2B and its titer was determined by fluorescent microscopy. The titer of the recombinant lentiviruses was approximately 2 × 10^9^ TU/ml.

### Inhibition of CVB3 replication in HeLa cells

**Figure 1:**
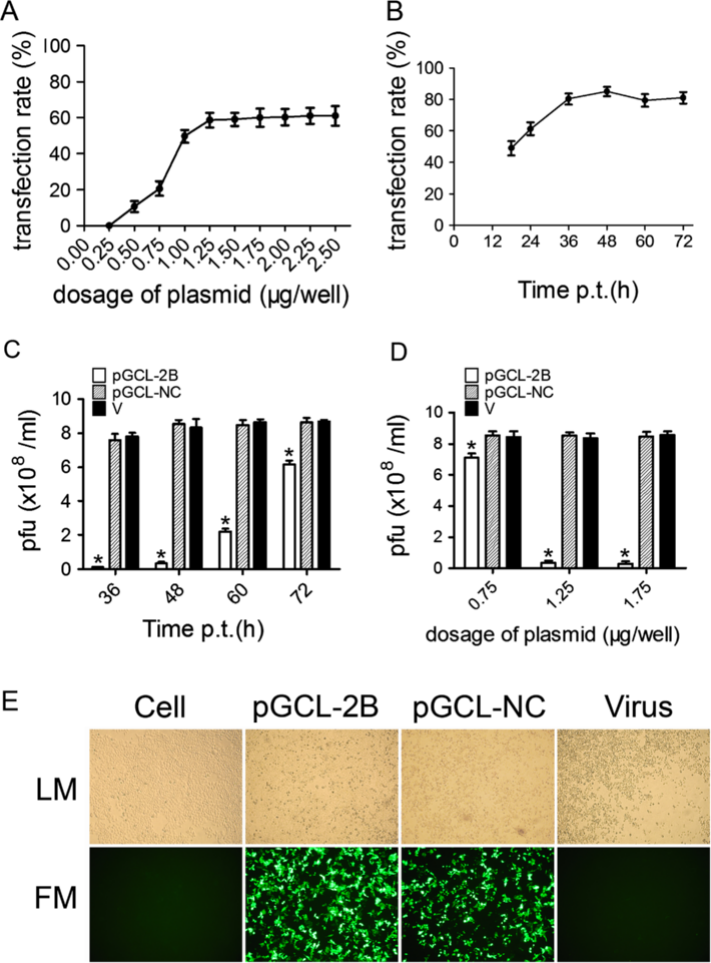
**Evaluation of the antiviral effects of pGCL-2B in HeLa cells.A** HeLa cells were pretreated with pGCL-2B at series of doses and the percent of GFP-expressing cells were counted through fluorescence microscopy after 24 h. The results of three independent experiments are expressed as mean ± standard deviation. **B** HeLa cells were transfected with pGCL-2B (1.25 μg/well), and the transfection rates were counted at different time points. The results of three independent experiments are expressed as mean ± standard deviation. **C** HeLa cells were transfected with pGCL-2B (1.25 μg/well) or pGCL-NC or MEM only (V), followed by CVB3 infection (MOI = 0.01). The viral titers were measured at 36, 48, 60, 72 h p.i. using supernatants of cell cultures by plaque-forming analysis and the results of three independent experiments are expressed as mean ± standard deviation. Significant differences between pGCL-2B treated (white bars) and pGCL-NC treated samples (striped bars) are indicated (*: *P* < 0.05) **D** HeLa cells were transfected with pGCL-2B at 0.75, 1.25 or 1.75 μg/well, respectively, or pGCL-NC or MEM only (V), followed by CVB3 infection (MOI = 0.01). The viral titers were measured at 48 h p.i. using supernatants of cell cultures by plaque-forming analysis and the results of three independent experiments are expressed as mean ± standard deviation. Significant differences between pGCL-2B treated (white bars) and pGCL-NC treated samples (striped bars) are indicated (*: *P* < 0.05) **E** HeLa cells were transfected with pGCL-2B (1.25 μg/well) or pGCL-NC or MEM only (Virus), followed by CVB3 infection (MOI = 0.01). After 48 h, GFP-expressing cells were visualized by fluorescence microscopy. *LM*: light microscopy, *FM*: fluorescence microscopy.

To evaluate the transfection efficacy of pGCL-2B, HeLa cells were transfected with pGCL-2B at a series of doses and the GFP-expressing cells were visualized through fluorescence microscopy after 24 h. The transfection rate of pGCL-2B was determined by the percent of the area of GFP-expressing cells versus that of whole HeLa cells in one field calculated using Anymicro DSS™ digital shoot system (Beijing Yutianshijiweiye Technology Development Co. Ltd., China). Figure 1A shows that 1.25 μg/well was the lowest dose of pGCL-2B to reach about 60% of cells expressing GFP and was less toxic to the cells. Higher the dose of plasmid used, more vacuolar change occurred in the cells. When HeLa cells were transfected with pGCL-2B of 6.25 μg/well, the cells died after 24 h. Subsequently, pGCL-2B of 1.25 μg/well was used to transfect HeLa cells, and GFP expressions detected at different time. After 48 h, GFP expression rate reached to the peak (Figure 1B). The transfection rate of control plasmid was similar to that of pGCL-2B. We also studied its antiviral efficacy as a function of time and dose in HeLa cells. Compared to the viral titer of pGCL-NC, an approximate 96% decrease in viral titer was detected in these cultures treated with pGCL-2B (1.25 μg/well) at 48 h post challenge. This protective effect was reduced after 60 h and 72 h (Figure 1C). Determination of CVB3 titers 48 h after infection showed a strong antiviral effect of the pGCL-2B transfection with titers reduced 95.83% at the plasmid dose of 1.25 μg/well and 96.46% at the plasmid dose of 1.75 μg/well (Figure 1D). Expectedly, HeLa cells pre-treated with a dose of 1.25 μg/well of pGCL-2B were efficiently protected against CVB3-induced cell lysis at 48 h after infection, while strong CPE occurred in other two groups (Figure 1E).

**Figure 2:**
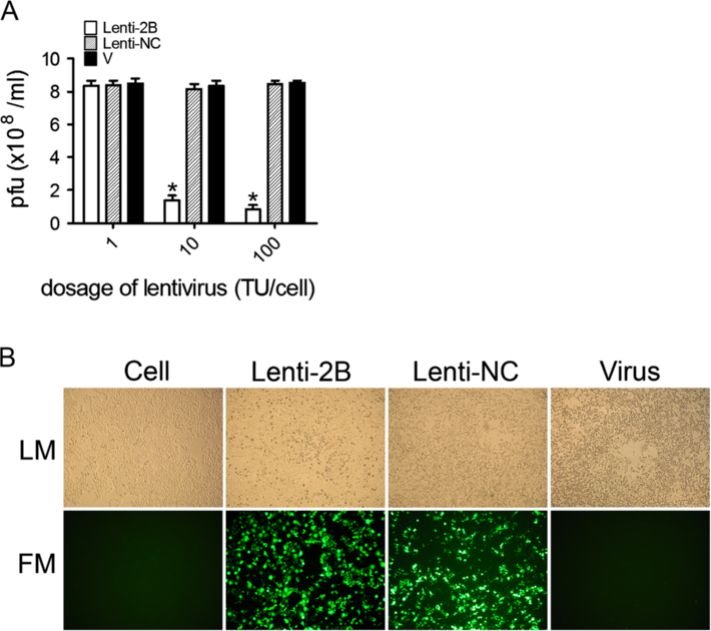
**Evaluation of the antiviral effects of Lenti-2B in HeLa cells.A** HeLa cells were transduced with Lenti-2B at 1, 10, 100 TU/ml, respectively, or Lenti-NC or MEM only (V), followed by CVB3 infection (MOI = 0.01). The viral titers were measured at 48 h p.i. using supernatants of cell cultures by plaque-forming analysis and the results of three independent experiments are expressed as mean ± standard deviation. Significant differences between Lenti-2B treated (white bars) and Lenti-NC treated samples (striped bars) are indicated (*: *P* < 0.05) **B** HeLa cells were transduced with Lenti-2B (10 TU/well) or Lenti-NC or MEM only (Virus), followed by CVB3 infection (MOI = 0.01). After 48 h, GFP-expressing cells were visualized by fluorescence microscopy. *LM*: light microscopy, *FM*: fluorescence microscopy.

To evaluate the efficacy of Lenti-2B on coxsackieviral replication, Lenti-2B (1, 10, 100 TU/cell) or Lenti-NC were used to transduce HeLa cells respectively. After 48 h, cells were infected with CVB3 (MOI = 0.01), and then the culture supernatants were collected to determine the CVB3 titers by viral plaque analysis at 48 h post challenge. Lenti-2B at a dose of 10 TU/cell or 100 TU/cell achieved an approximately 84% and 90%, respectively, reduction of CVB3 titer compared with that of control cells treated with Lenti-NC. The antiviral effect was almost absent at the dose of 1 TU/cell because of the low transduction efficacy of the lentivirus (Figure 2A). Lenti-2B also exhibited the protective effect against CPE (Figure 2B). These results indicate that recombinant plasmid and lentivirus expressing shRNA-2B are highly efficient antiviral agents *in vitro*.

### Inhibition of CVB3 replication in coxsackievirus-induced myocarditis model

**Figure 3:**
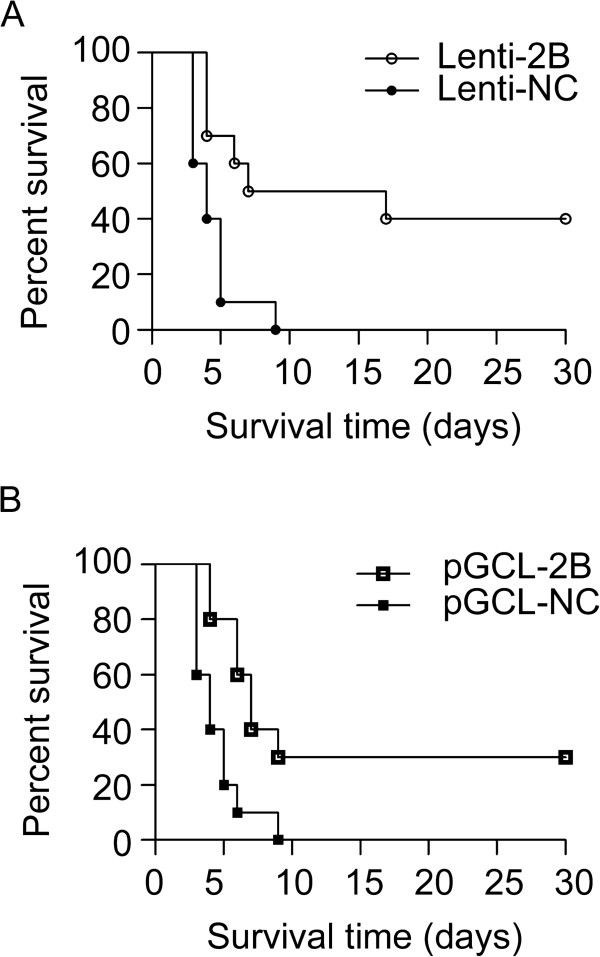
**Evaluation of the antiviral effects of shRNA-2B on survival rate.** BALB/c mice (n = 10) were injected with plasmid (40 μg/mouse) or lentivirus (1 × 10^8^ TU/mouse) through a tail vein, 24 h, post injection, 8 × 10^3^ pfu/mouse of CVB3 was used as the challenge.

To reveal the effect of shRNA-2B on survival, virus replication, and tissue damage, pGCL-2B (20 μg/mouse, 30 μg/mouse or 40 μg/mouse) or Lenti-2B (1 × 10^7^ TU/mouse, 5 × 10^7^ TU/mouse or 1 × 10^8^ TU/mouse) was injected into mice before infection with CVB3 virus. The control vector pGCL-NC or Lenti-NC was applied in the same way. Transfection of pGCL-NC or Lenti-NC had no effect on survival compared with the infected but non-treated animals. All mice died in 9 days after infected with CVB3. In contrast, in the group infected with 1 × 10^8^ TU/mouse Lenti-2B, 50% mice were still alive at day 9 and 40% mice lived at 30 days after challenge with CVB3 (Figure 3A). The pGCL-2B group (40 μg/mouse) also showed a higher survival rate than that of the pGCL-NC control group (30% vs. 0 survival rate, *P* < 0.01) at day 9 post CVB3 infection (Figure 3B). However, transfection of pGCL-2B or Lenti-2B with lower doses had no difference on survival between treated group and control group. Both pGCL-2B and Lenti-2B improved significantly the life span of mice, suggesting that shRNA-2B could be the potential agent to reduce the mortality caused by viral myocarditis.

**Figure 4:**
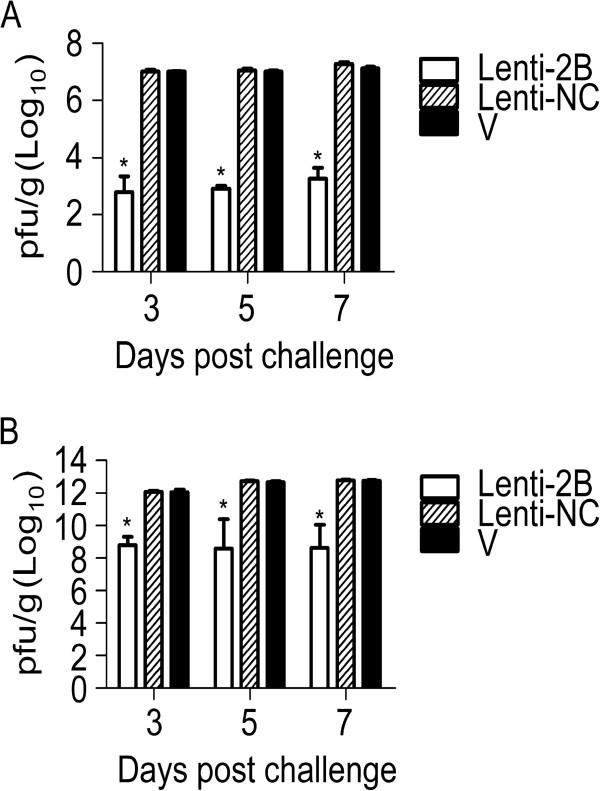
**Antiviral effects of Lenti-2B on the viral titers in the hearts (A) and pancreases (B) of mice infected with CVB3.** BALB/c mice were pretreated with lentiviruses expressing shRNAs under the condition described in the legend to Figure 3, and euthanized at days, 3, 5 and 7 post infection with CVB3 (n = 5). The heart and pancreas tissues were weighted and made a 0.2% (w/v) homogenate in 0.5 ml MEM, followed by the determination of the viral titers. Histogram represents average values of experiments performed with samples obtained from five different mice. Error bars represent mean ± standard deviation. *P < 0.01.

**Figure 5:**
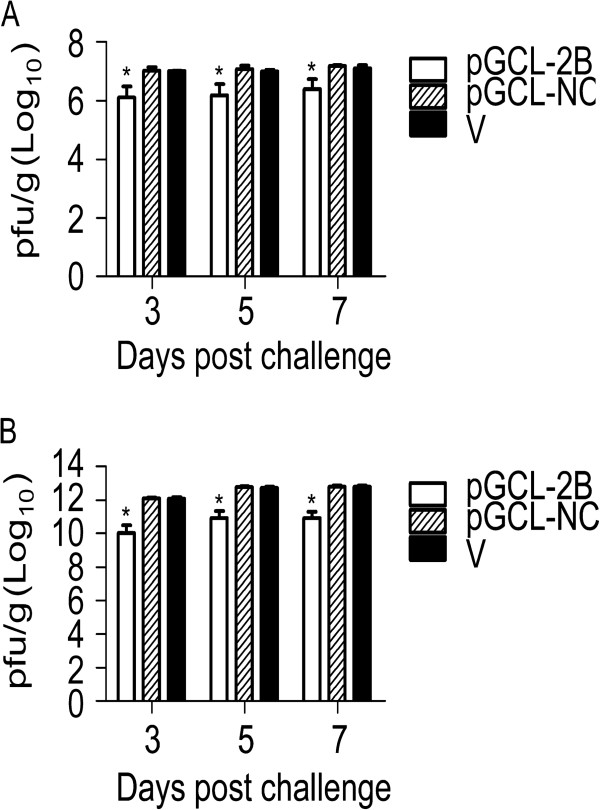
**Antiviral effects of pGCL-2B on the viral titers in the hearts (A) and pancreases (B) of mice infected with CVB3.** BALB/c mice were pretreated with plasmid expressing shRNAs under the condition described in the legend to Figure 3, and euthanized at days 3, 5 and 7 after infection with CVB3 (n = 5). The heart and pancreas tissues were weighted and made a 0.2% (w/v) homogenate in 0.5 ml MEM, followed by the determination of the viral titers. Histogram represents average values of experiments performed with samples obtained from five different mice. Error bars represent mean ± standard deviation. *P < 0.01.

To further determine the potency of Lenti-2B, mice were euthanized to directly compare the tissue virus titers by plaque-forming assay at days 3, 5 and 7 post CVB3 infection. The virus titers in the hearts and pancreases of mice infected with Lenti-2B were approximately 4-log_10_ lower than those in the control group (*P* < 0.01 versus Lenti-NC group for both organs, n = 5 in each group) (Figure 4). Virus titers were 1-log_10_ lower in the heart and 2-log_10_ lower in the pancreas of mice that received pGCL-2B than that in the respective control mice (Figure 5). Furthermore, the virus titer was 10^3^-fold lower in the heart of Lenti-2B group than that of pGCL-2B group, suggesting that lentivirus vector expressed shRNA-2B more effectively than plasmid *in vivo* (*P* < 0.01).

**Figure 6:**
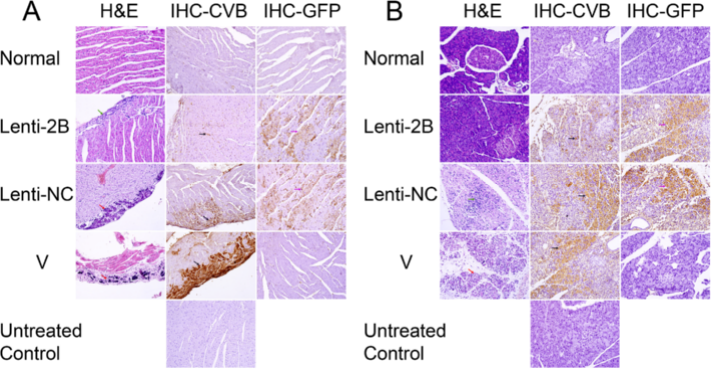
**Histology of myocardial inflammation (A) and pancreatic inflammation (B) at day 7 after CVB3 challenge.** BALB/c mice were injected with Lenti-2B (1 × 10^8^ TU/mouse) through the tail vein, at 24 h post injection, 8 × 10^3^ pfu/mouse of CVB3 was used as the challenge, and then euthanized at day 7 post CVB3 infection. The four groups were injected with Lenti-2B or Lenti-NC or MEM but infected with CVB3 (V) or MEM only as normal control. Hematoxylin-eosin staining and immunohistochemical analysis of CVB3 or GFP were performed on the sections prepared from the hearts. *Untreated Control* shows the control section was treated without the first antibody in IHC staining. *Green arrow* shows a example of infiltration, *red arrows* show examples of necrosis and calcification, *black arrows* show examples of CVB3 infection, *purple arrows* show examples of GFP-expressing. Magnification, 200×.

To confirm the protect effect of Lenti-2B on the hearts and pancreases of CVB3 infected mice, hematoxylin-eosin staining was used to detect inflammation and immunohistochemistry staining was used to detect the distribution of CVB3 viruses or GFP expression in the organs. No, or only slight, myocardial inflammation and CVB3 infection were revealed in the Lenti-2B group; however, there were large extent of necrosis, calcification and serious virus infection in the Lenti-NC group and non-shRNA-treated group (Figure 6).

## Discussion

Coxsackievirus infection can injure cell directly and damage various tissues, leading to viral heart disease [2], pancreatitis [3], or meningitis [4]. At present, there is no specific antiviral therapy available [21]. RNAi-based antiviral therapy has potential to degrade viral RNA and promote viral clearance [22]. In previous publications, RNAi was applied to inhibit coxsackievirus replication by siRNAs targeting viral 2A [23,24], 3D [25], and VP1 [15,25]. These synthetic siRNAs were tansfected into mammalian cells or delivered into mice via the tail vein by high-volume injections and protected cells or organs from virus-mediated injury. However, the degradation of siRNAs by intracellular nucleases and the method of delivery limited the effect *in vivo*. To overcome some limitations of siRNAs in antiviral applications, adeno-associated virus-delivered shRNA directed at the RNA polymerase of CVB3 was reported to significantly attenuate the cardiac dysfunction [26] and lentivirus-delivered shRNA against 2C was reported to improve survival rate in an animal mode [27]. It was suggested that shRNA viral vector could be used effectively to prevent coxsackievirus replication in mice.

In this study, RNA interference was employed to target the CVB3 protein 2B region (3753-3771), which is a highly conserved region in most enteroviruses and therefore is an attractive therapeutic target. Among twelve siRNAs we tested, siRNA-2B was proved most highly efficient in the inhibition of viral replication in HeLa cells. In subsequent animal experiments, lentivirus-delivered shRNA-2B was shown to improve the survival rate of 40% at 30 days after challenge. Furthermore, the viral titers in the mouse hearts and pancreas of the Lenti-2B group were significantly reduced compared with those of the control group. The biopsy of hearts and pancreas of mice injected with Lenti-2B also showed less inflammation, suggesting that shRNA-2B effectively inhibits viral replication and reduces the virus-induced myocarditis in the animal model. This study indicated that shRNA-2B can reduce CVB3 progeny production by cleavage of viral genomic RNA, which results in the suppression of viral particle assembly with intact CVB3 RNAs and hampering of the whole translation process of the viral polyprotein. On the other hand, it was reported that 2B protein plays a major role in suppressing apoptotic host cell response by manipulating intracellular Ca^2+^ homeostasis and, thereby, in extending the life span of the host cell [28]. However, this RNAi by shRNA-2B may enhance host cell death and subsequently limit CVB3 replication. Therefore, shRNA-2B inhibits CVB3 replication not only directly through RNAi but also indirectly through enhancing host cell death.

In recent years, many studies have reported successful inhibition of viral replication in cultured cells or in murine models using transient transfection of synthetic siRNA or plasmid expressing shRNA. However, the antiviral effect of RNAi depends on the delivery efficacy of siRNA or shRNA. We evaluated the effect of shRNA-2B expressed using both plasmid vector and lentiviral vector. The data presented here indicate that the viral titers in HeLa cell supernatants were less in the pGCL-2B group than in the Lenti-2B group. Inversely, the viral titers in mouse heart and pancreas were decreased much more in the Lenti-2B group than in the pGCL-2B group, suggesting that the expression of shRNA-2B with viral vector exhibited higher inhibitory efficiency on viral replication than with plasmid vector *in vivo*. Thus, lentiviral vector is a promising viral vehicle for delivery of shRNAs and shRNA-2B has great potential to be further developed into an effective therapeutics for the treatment of coxsackievirus and other enterovirus infections.

## Conclusions

shRNA-2B significantly reduced the virus titer of CVB3 in HeLa cells, also prolonged the mouse survival span, attenuated the tissue damage and inhibited the viral production *in vivo* compared with those of the shRNA-NC treated control group, suggesting that shRNA-2B is a potentially therapeutic agent for the treatment of enterviral diseases.

## Abbreviations

CVB3: Coxsackievirus B3; RNAi: RNA interference; shRNA: Short hairpin RNA; dsRNA: Short double-stranded RNA; siRNA: Small interfering RNA; DMEM: Dulbecco’s modified Eagle’s medium; PBS: Phosphate buffered saline; GFP: Green fluorescent protein.

## Competing interests

The authors declare that they have no competing interests.

## Authors’ contributions

HY carried out most experiments and drafted the manuscript. FH carried out the animal experiments. CW measured virus titers in cells. ZX & JZ evaluated pathology. FW performed the statistical analysis. YZ & ZL conceived of the study, and participated in its design and revised the manuscript. All authors read and approved the final manuscript.

## Pre-publication history

The pre-publication history for this paper can be accessed here:

http://www.biomedcentral.com/1471-2334/12/177/prepub
